# L3MBTL3 is induced by HIF-1α and fine tunes the HIF-1α degradation under hypoxia in vitro

**DOI:** 10.1016/j.heliyon.2023.e13222

**Published:** 2023-01-24

**Authors:** Mengdong Wang, Di Wang, Yue Lang, Anwen Shao, Rui Zhang, Jun Tang, Dongming Lai, Chenglu Xiao

**Affiliations:** a National Key Laboratory of Veterinary Public Health Security, College of Veterinary Medicine, China Agricultural University, Beijing, 100193, China; b Sun Yat-sen Memorial Hospital, Sun Yat-sen University, NO. 107, Yanjiangxi Road, Guangzhou, 510120, China

**Keywords:** L3MBTL3, HIF-1α, Hypoxia, HIF-1α degradation, **HIF-1**, hypoxia inducible factor 1, **ARNT**, aryl hydrocarbon receptor nuclear translocator, **L3MBTL3**, lethal (3) malignant brain tumor-like 3, **MBT**, malignant brain tumor, **PHD**, prolyl hydroxylase domain, **VHL**, von Hippel-Lindau, **HRE**, hypoxia response element, **CHX**, cycloheximide, **FCS**, phenylalanine-cysteine-serine nucleic acid−binding, **SAM**, sterile α motif

## Abstract

HIF-1α plays a crucial part in hypoxia response by transcriptionally upregulating genes to adapt the hypoxic condition. HIF-1α is under severe cellular control as its exceptional activation is always associated with tumorigenesis and tumor progression. Here, we report L3MBTL3 serves as a novel negative regulator of HIF-1α. It is upregulated during hypoxia and acts as a transcriptional target of HIF-1α. In the nuclei, L3MBTL3 makes an interaction with HIF-1α and promotes its ubiquitination and degradation. These findings indicate L3MBTL3 forms a negative feedback loop with HIF-1α in vitro to dampen the hypoxic response.

## 1 Introduction

Hypoxia inducible factor 1 (HIF-1) is a pivotal mediator in hypoxia response which plays a crucial part in promoting cancer metastasis, and participates in the process of apoptosis, angiogenesis and proliferation [[1], [2], [3], [4]]. HIF-1 belongs to a kind of heterodimeric transcription factor composing two subunits, namely HIF-1α and HIF-1β, also referred as ARNT [5].

HIF-1 is activated in response to hypoxia, mainly due to a rapid increase in the cellular level of HIF-1α resulted from its stabilization [6]. Under normoxia, the amount of HIF-1α in cells is extremely low as the conserved proline residues in HIF-1α are hydroxylated by O_2_ sensitive PHD proteins. The hydroxylated HIF-1α is then recognized by the E3 ligase Hippel-Lindau (VHL) resulting in its rapid ubiquitination and degradation [7,8]. Under hypoxia, the hydroxylation of HIF-1α is reduced. HIF-1α is stabilized, and its cellular level is increased significantly. The stabilized HIF-1α is transferred into the nucleus and attaches to HIF-1β [9]. The HIF-1α/1β complex then binds to the hypoxia response element (HRE) of its target genes, and further recruits the co-activator p300/CBP to promote gene transcriptions critical for hypoxic adaption [4,10,11].

Studies have shown that sustained HIF-1α activation promotes tumor-associated angiogenesis, as well as tumor cell survival and proliferation [4,12,13]. To avoid the detrimental consequence, HIF-1 signaling is under strict regulation. The HIF-1 signaling can be downregulated by degradation of the hypoxic HIF-1α and/or suppression of its transactivation activity. PHD2 and PHD3 are both the key regulators essential for downregulation of HIF1 in different systems [14,15]. Multiple oxygen independent degradation pathways of HIF-1α have been reported, including VHL-dependent and independent mechanisms [16]. For instance, it is reported that the HIF-1α methylation by SETD7/9 can lead to its degradation through 26 S proteasomes, independently of HIF-1α hydroxylation [17].

L3MBTL3, also known as MBT1, acts as a member of the MBT (malignant brain tumor) family [18]. L3MBTL3 was previously isolated as a PcG protein in mammals and is highly expressed in hematopoietic progenitor cells. The mice with null mutation of L3MBTL3 displayed embryonic lethality due to anemia [19]. L3MBTL3 contains three repeated MBT domains which function as a mono- or demethylated lysine reader to histones [20,21]. It is reported that L3MBTL3 is also involved in methylation mediated protein degradation. It has been shown that L3MBTL3 bound methylated DNMT1, E2F or SOX2 and recruited a ubiquitin ligase to degrade these proteins [22,23]. Here we provide evidence to suggest that L3MBTL3 also regulates the stability of HIF-1α in vitro. We demonstrated that in response to hypoxia, the transcription of L3MBTL3 is increased substantially by HIF-1α. Then the up-regulated L3MBTL3 unites to HIF-1α and promotes its degradation through ubiquitination.

## 2 Materials and methods

### 2.1 Constructs and reagents

Full-length HIF-1α and L3MBTL3 was cloned from HeLa cell lines. After that, *N*-terminal HA tagged human whole HIF-1α and FLAG tagged human whole L3MBTL3 coding sequences were ligated into pRK5 vector. The constructions of plasmids were achieved by homologous recombinant, and the restriction sites of pRK5 vector was *Bam*HI and *Hin*dIII. The plasmids oligos were synthesized by Tsingke (Beijing,China) as follows:

HA–HIF–1α forward:

5′-GACTATGCGGGCGGATCCATGGAGGGCGCCGGCGGCGCG-3′

and reverse:

5′-AGTTGGGCCATGGCGGCCAAGCTTTCAGTTAACTTGATCCAAAGC-3’.

Flag-L3MBTL3 forward:

5′-AAGGACGACGATGACAAGGGATCCATGACTGAATCTGCCTCT-3′

and reverse:

5′-AGTTGGGCCATGGCGGCCAAGCTTTCAAAGTTCATTGTGAGA-3’.

The point-mutation and truncation mutants of Flag-L3MBTL3 were constructed by PCR and cloned into the pRK5 vector.

The L3MBTL3 promoter sequence (from −1500 to 1000) was ligated to the pGL3-Basic vector (Promega, USA).

Immunoblot analysis using the antibodies: *anti*–HIF–1α (36169 S, CST, 1:1000), *anti*-PGK1 (ab113687, Abcam, 1:10,000), *anti*-L3MBTL3 (ab68117, Abcam, 1:10,000), *anti*-L3MBTL3 (K005142P, Solarbio, 1:1000), *anti*-α-Tubulin (PM054, MBL, 1:10,000), anti-Flag (F1804, Sigma, 1:1000), and anti-HA (SC-805, Santa Cruz Biotechnology, 1:1000).

Cycloheximide (CHX) and MG132 were purchased from Sigma.

### 2.2 Cells and transfection

HeLa, HepG2, H1299, and HEK293T cell lines originated in the American Type Culture Collection (Manassas, VA, USA) were cultured in Dulbecco's minimum essential medium (DMEM) supplemented with 10% (v/v) fetal bovine serum (FBS, Invitrogen) and penicillin (100 U/mL, Invitrogen)-streptomycin (100 μg/mL, Invitrogen). All cells were cultured in the usual condition, namely maintained in 5% CO_2_ at 37° Celsius. Hypoxia condition was performed in modular incubator chamber with 1% O_2_.

Cells were applied with jet PRIME (Polypus-transfection SA) to transfect plasmid transiently according to the manufacturer's protocol. Using Lipofectamine RNAiMax (Invitrogen) mediated siRNAs delivery to inhibit the target gene expression.

HIF-1α RNAi sequence has previously been described [24]. The target sequences of control siRNA and L3MBTL3 siRNA target sequences were designed by The Genetic Perturbation Platform (https://portals.broadinstitute.org/gpp/public/). The siRNA oligos were synthesized by GenePharma (Shanghai, China) as follows:

siCtrl, 5′-TTCTCCGAACGTGTCACGT-3′,

siL3MBTL3, 5′-CAATCGTTTCCTGGTACATTT-3’.

To generate the stable gene (L3MBTL3) knock-out cell line, as described previously [25], HeLa cells that reached seventy percent confluency were transfected with Cas9 plasmids containing a guide RNA target sequence of third exon on L3MBTL3 (gRNA: 5′-GCTCTTGACCACTTGTGCTAG-3′). At 48 h post transfection, diluted the cells and seeded them into a 96-well dish (0.5 cell per well) with DMEM media. Then selected the single colony to expand for Western blot analysis of L3MBTL3.

### 2.3 Real-time PCR

To obtain the purified total mRNA, TRIzol reagent (Invitrogen) was employed according to the manufacturer's protocol. *M*-MLV reverse transcriptase (Promega) was used to reverse transcribe total RNA (about 0.8 μg from each sample) to get the cDNA. The cDNA and primers were then mixed with UltraSYBR Mixture (Beijing CoWin Biotech) according to the protocol and loaded to Applied Biosystems ViiA 7 Real-Time PCR System. This analysis employed the following primers.

**Table:** 

Gene	Forward (5′-3′)	Reverse (5′-3′)
L3MBTL3	TCCTGAGCATCAGTCTGTGTA	AGAGCGTCTGCATTCACCC
PGK1	TGGACGTTAAAGGGAAGCGG	GCTCATAAGGACTACCGACTTGG
HIF-1α	GAACGTCGAAAAGAAAAGTCTC	CCTTATCAAGATGCGAACTCACA
GAPDH	AGCCAAAAGGGTCATCATCTC	GGACTGTGGTCATGAGTCCTTC

GAPDH is the endogenous control.

### 2.4 Western blot, immunofluorescence, immunoprecipitation and ubiquitination analysis

For Western blot analysis, collected cells were lysed in the lysis buffer (50 mM Tris-Cl at pH 8.0, 150 mM NaCl, 1% Triton X-100, 10 mM DTT, 1 × complete protease inhibitor cocktail pill and 10% glycerol) as described previously [25]. The lysates were loaded to SDS-PAGE gel and then transferred onto PVDF membranes. The membranes were then blotted with indicated antibodies. Immobilon classico Western HRP substrates (212,885, Millipore) were added onto PVDF membrane for reaction, after 2–5 min, the images were developed in the Tanon-5200 automatic chemiluminescence image analysis system. The relative intensity of the bands was analyzed by ImageJ.

For Immunofluorescence analysis, cells cultured on glass coverslips were transfected with plasmids. After 12 h treatment in incubator chamber with 1% O_2_, the cells were fixed with 4% paraformaldehyde for 10 min, permeabilized with 0.2% Triton X-100 for 5 min on ice and blocked with 1% BSA (bovine serum albumin) for 45 min. The cells were then incubated with the HIF-1α antibody (36169 S, CST, 1:200) and L3MBTL3 antibody (ab68117, Abcam, 1:200), followed by a TRITC-conjugated goat anti-mouse secondary antibody and FITC-conjugated goat anti-rabbit secondary antibody incubation. Nuclei were counterstained with 4′,6′-diamidino-2-phenylindole (DAPI; Beyotime, 1:1000). The images were captured using a Nikon H600L microscope.

To do the immunoprecipitation analysis, indicated cells were treated with or without MG132 for 6 h then lysed in the lysis buffer. Then centrifuged the lysate at low temperature (4 °C) for 20 min at maximum speed. Anti-Flag M2 beads (5 μl, Sigma) or the referential antibodies (1 μg) and protein G (GE) were added to the supernatants, and rotated for 6 h at 4 °C. Nuclear and Cytoplasmic Protein Extraction Kit (Beyotime Biotechnology) was used for nuclear and cytoplasmic separation.

For ubiquitination analysis, 293 T cells were co-transfected with Ub, HIF-1α and L3MBTL3 plasmids, as indicated. The cells were cultured under hypoxic state for one day and night with MG132 inhibitor treated for 6 h. Then cells were lysed in 80 μl lysis solution and 10 mM NEM were added before used, cell lysates were boiled for 10 min at 95 °C, diluted with 10 vol of lysis buffer, and then centrifuged at maximum speed for 15 min at 4 °C. The anti-Flag antibody was added to supernatants. The mixture was incubated and rotated at 4 °C for at least 6 h.

### 2.5 GST-pull down

5 mL E*. coli* induced by Isopropyl β-D-Thiogalactoside (IPTG, Sigma) was centrifuged and resuspended in 200 μl cold PBS buffer. The resuspending was then conduct sonication treatment (10 min). The lysates were then centrifuged at 12,000 rpm for half an hour (4 °C). 5 μl Glutathione Sepharose (GE) was added to the supernatants and then incubated with rotation at low temperature for 1 h. The washed Glutathione Sepharose (rinsed for 3 times) was then incubated with cell lysates containing HA-L3MBTL3 at 4 °C for 6 h.

### 2.6 Luciferase reporter assay

Luciferase reporter assay was performed as described previously [24]. Briefly 293 T cells were transfected with 100 ng luciferase reporter plasmids, 20 ng pRL-TK Renilla plasmids served as internal reference and the indicated plasmids. Luciferase activity was measured using the Dual-Luciferase Reporter Assay System Kit (Promega).

### 2.7 Statistical analysis

**Fig. 1:**
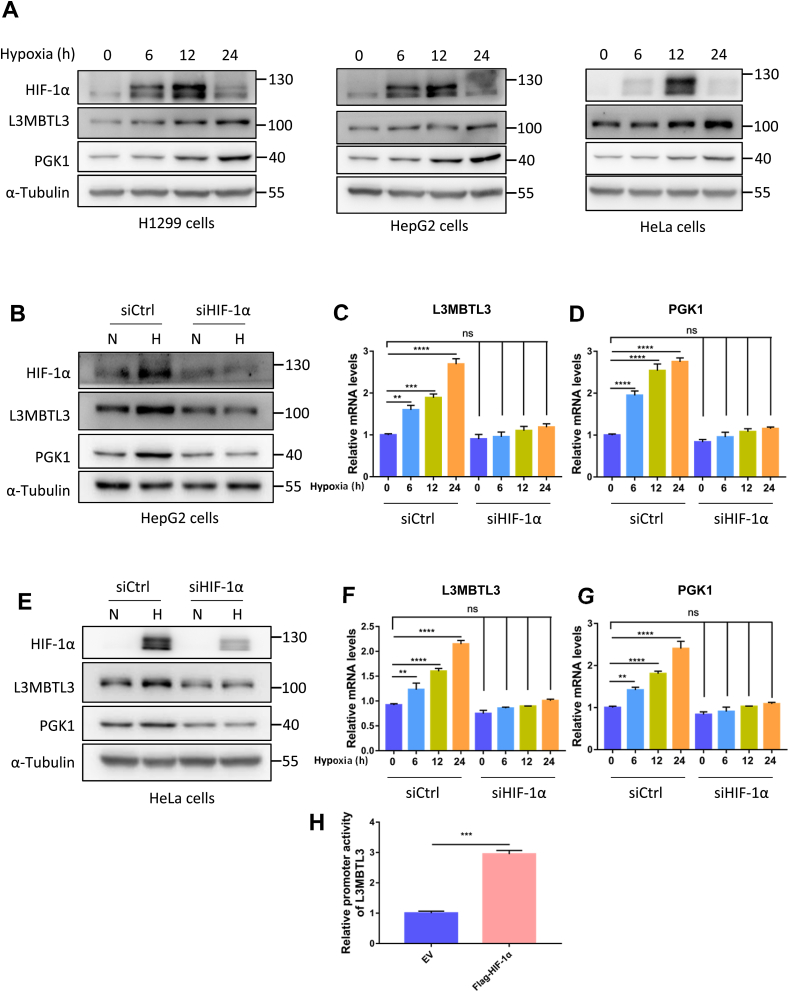
Hypoxic conditions induces L3MBTL3 upregulation A. The protein level of L3MBTL3 was upregulated under hypoxia. H1299, HepG2 and HeLa cells were treated with low oxygen conditions (1% O_2_) for the displayed time points. The cells lysates were analyzed by Western blot using the indicated antibody. The experiment was repeated at least once. B-G. L3MBTL3 upregulation caused by low oxygen conditions was suppressed by reduction of HIF-1α. In respect of normoxia or hypoxia, Western blot or real-time PCR analysis of HepG2 (B, C and D) and HeLa (E, F and G) cells transfected with siRNA of control or HIF-1α. H. HIF-1α enhanced L3MBTL3 promoter activity. Data are shown as mean ± SD of three independent experiments. Statistical analysis was performed by the one-way ANOVA test followed by Dunnett's multiple comparisons test (C, D, F and G) or the Student's *t*-test (H). **p < 0.01; ***p < 0.001; ****P < 0.0001. N = 2, the N numbers indicated biological replicates. The raw figures for Western blot were shown in the Supplementary Figure S4.

GraphPad Prism was used for the statistical analysis, and the analysis of variance (ANOVA) was performed on at least three independent replicates. P values of <0.05 were considered statistically significant for each test. *P < 0.05; **P < 0.01; ***P < 0.001; ****P < 0.0001; ns for non-significant. The source analysis data for Fig. 1C, D, 1F, 1G and 1H done by GraphPad Prism software were provided in the Supplementary Table 1.

## 3 Results

### 3.1 Hypoxic conditions induce L3MBTL3 upregulation

To study whether L3MBTL3 is regulated by hypoxic conditions. H1299, HepG2, and HeLa cell were treated with 1% O_2_ (hypoxia) for 6, 12 and 24 h respectively. Western blot analysis revealed that L3MBTL3 as well as PGK1, a known target of HIF-1α, were upregulated under hypoxic treatments in a time-dependent way (Fig. 1A, Supplementary Figure S1A, 1C). We then checked out whether the increase of L3MBTL3 was relied on HIF-1α. HIF-1α expression in HepG2 and HeLa cells was knocked down by siRNA. Both the Western blot and RT-PCR assays revealed that HIF-1α knockdown abolished the hypoxia induced upregulations of L3MBTL3 and PGK1 (Fig. 1B–G, Supplementary Figure S1B, 1D, 1E). To test the hypothesis that L3MBTL3 was a direct target of HIF-1α, L3MBTL3 promoter region (from −1500 to 1000 bp relative to the transcription start site) was ligated to a luciferase reporter plasmid. This reporter clone was then transfected into the 293T cells with either control vectors or plasmids expressing HIF-1α. The intensity of luciferase was enhanced by HIF-1α transfection (Fig. 1H). Collectively, above data demonstrate the phenomenon that L3MBTL3 is induced by hypoxic conditions dependent on HIF-1α and is a direct transcriptional target of HIF-1α in vitro.

### 3.2 L3MBTL3 downregulates the protein level of HIF-1α under hypoxia

**Fig. 2:**
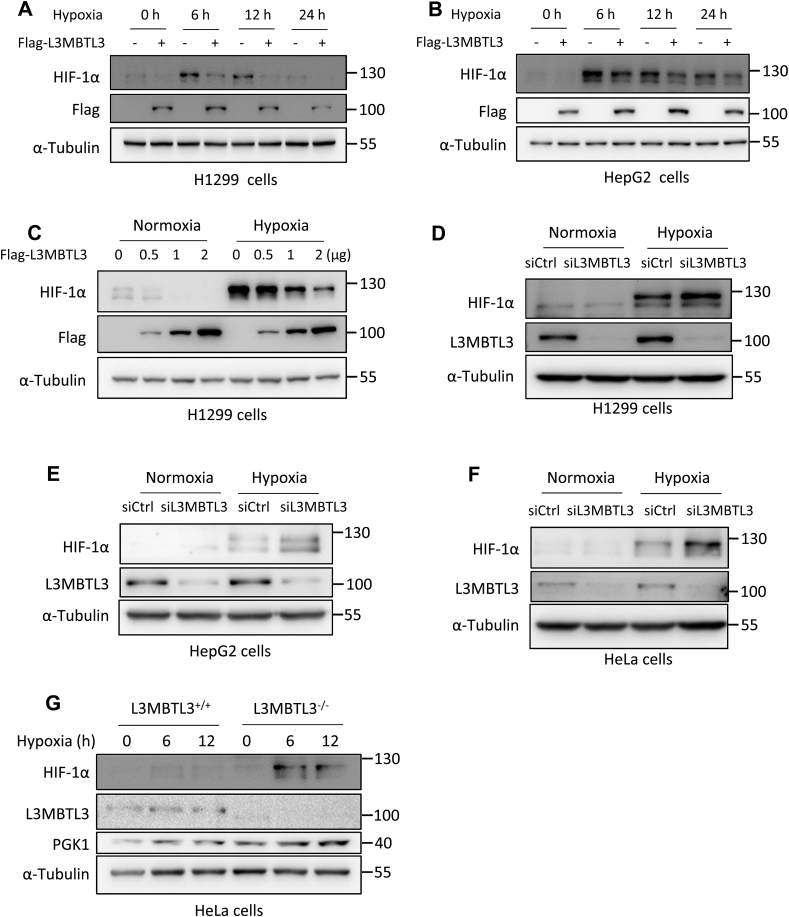
L3MBTL3 downregulates HIF-1α protein level in hypoxic condition A, B. L3MBTL3 reduced HIF-1α expression induced by low oxygen conditions. H1299 (A) and HepG2 (B) cells were transfected with control or Flag-L3MBTL3 plasmids, and then treated with hypoxia for the indicated time points. Western blot analysis was used to analyze the protein level in cell lysate. The experiment was repeated at least once in HepG2 cells. C. L3MBTL3 inhibited HIF-1α protein levels in a dose dependent manner. D-F. L3MBTL3 knockdown enhanced protein levels of HIF-1α upregulated by hypoxia. H1299 (D), HepG2 (E) and HeLa (F) cells transfected with siRNA of control or L3MBTL3 were culture in hypoxic conditions for 6 h. And then the cell lysates were gone through Western blot analysis. G. L3MBTL3 knock-out upregulated HIF-1α protein levels induced by hypoxia. The raw figures for Western blot were shown in the Supplementary Figure S4.

It is reported that L3MBTL3 plays a role in transcriptional repression and methylation-dependent protein degradation [18,22,23]. We then examined whether L3MBTL3 did regulate HIF-1α. We over-expressed L3MBTL3 in the H1299 and HepG2 cells and then incubated cells with hypoxia condition for 6, 12 and 24 h. The results from Western blot assay showed that HIF-1α was declined by L3MBTL3 overexpression (Fig. 2A and B, Supplementary Figure S2A, 2B, 2I). Dose dependent analysis in H1299 cells showed that L3MBTL3 downregulated HIF-1α protein levels in a dose dependent manner (Fig. 2C, Supplementary Figure S2C). L3MBTL3 was then knocked down employing siRNAs in H1299, HepG2 and HeLa cells. The cells were either untreated or treated by hypoxia. Western blot analysis displayed that L3MBTL3 knockdown enhanced the HIF-1α protein level caused by hypoxia, but it didn't affect the hypoxia untreated cells (Fig. 2D–F, Supplementary Figure S2D-F). Moreover, hypoxia induced HIF-1α and its target PGK1 upregulation were enhanced in L3MBTL3^−/−^ cells compared to control shown by Western blot (Fig. 2G, Supplementary Figure S2G-H). Together, these results hinted that L3MBTL3 downregulated the protein level of HIF-1α under hypoxia in vitro.

### 3.3 L3MBTL3 regulates HIF-1α stability under hypoxia in vitro

**Fig. 3:**
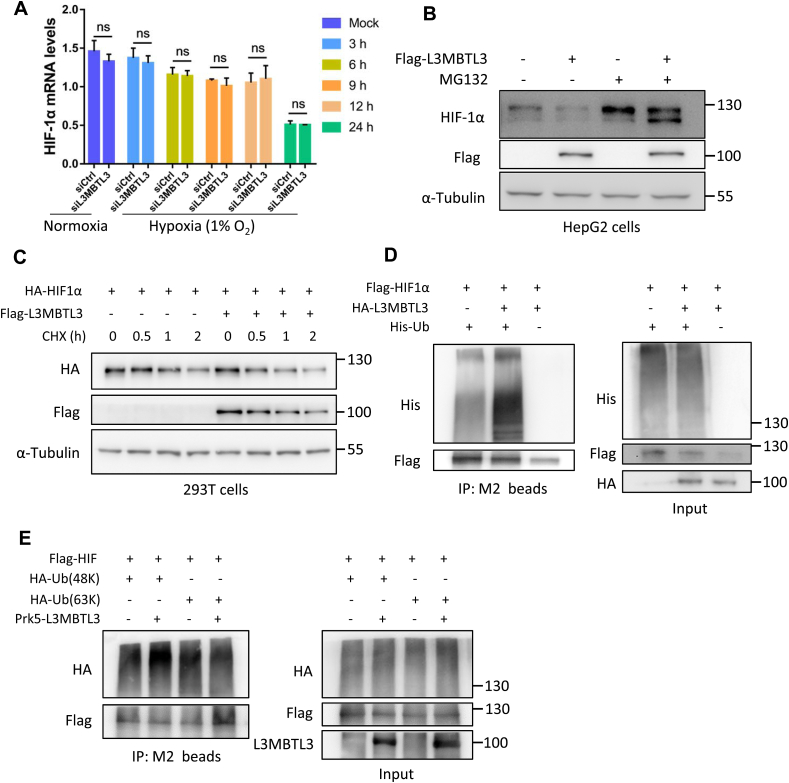
L3MBTL3 regulates HIF-1α stability under hypoxia in vitro A. L3MBTL3 knockdown did not influence the transcription of HIF-1α no matter in normoxia or hypoxia conditions. Data are shown as mean ± SD of three independent experiments. Statistical analysis was performed by the two-way ANOVA test. **p < 0.01; ***p < 0.001; ns for non-significant. B. L3MBTL3 overexpression mediated instability of HIF-1α was abolished by MG132 inhibition. C. L3MBTL3 reduced the HIF-1α stability. 293T cells were transfected with control or Flag-L3MBTL3 plasmids, and then cultured under hypoxia for 6 h followed by treatment with cycloheximide inhibitor for 0 h, 0.5 h, 1 h or 2 h. The cell lysates were analyzed by Western blot. D. L3MBTL3 enhances HIF-1α ubiquitination. Hypoxic conditions cultured 293T cells transfected with control vector or HA-L3MBTL3 constructs together with Flag–HIF–1α and His-Ub plasmids, 20 h after transfection, the cells were treated with MG132 inhibitor for additional 4 h. Then cell lysates were immunoprecipitated with the anti-Flag (M2) beads. An anti-His antibody was used to explore the HIF-1α ubiquitination. E. L3MBTL3 enhances K48-linked polyubiquitination of HIF-1α. Hypoxia cultured 293T cells co-transfected with Flag–HIF–1α, HA-Ub (R48K) or HA-Ub (R63K) and Prk5-L3MBTL3 plasmids were also treated with MG132 as above. Denatured cell lysates were subjected to Western blot analysis. An anti-HA antibody was employed to do the ubiquitination analysis. The raw figures for Western blot were shown in the Supplementary Figure S4.

We further investigated how L3MBTL3 downregulated HIF-1α. We evaluated the impression of L3MBTL3 knockdown on HIF-1α mRNA expression level. The real-time qPCR data showed that HIF-1α mRNA levels were not affected when L3MBTL3 was knocked down by siRNA during hypoxia (with 1% O_2_) (Fig. 3A). To figure out whether L3MBTL3 negatively regulates HIF-1α via ubiquitin-proteasome mediated degradation, we transfected HepG2 cells with the control or Flag-L3MBTL3 plasmids. The transfected cells were then cultured under hypoxia for 6 h, followed by treating with or without the MG132 proteasome inhibitor. Western blot analysis indicated that treatment of MG132 nearly abolished the repressive affection of Flag-L3MBTL3 on protein levels of HIF-1α (Fig. 3B). To test if L3MBTL3 affects the HIF-1α stability, the half-life of HIF-1α protein was conducted to analyze. 293T cells with Flag-L3MBTL3 or control plasmids were transfected with the HA–HIF–1α plasmids and cultured under hypoxia for 6 h, then followed by treating with the CHX inhibitor for 0, 0.5, 1 or 2 h. Compared with control cells, Flag-L3MBTL3 expressing cells exhibited a shortened half-life of HA–HIF–1α (Fig. 3C, Supplementary Figure S3A). To inquire into whether L3MBTL3 promotes HIF-1ɑ degradation was relied on ubiquitination, 293T cells were transfected with control vectors or HA-L3MBTL3 plasmids together with Flag–HIF–1α and His-Ub plasmids, and then treated with MG132 cultured in hypoxia. Cells expressing HA-L3MBTL3 displayed an enhanced ubiquitination of Flag–HIF–1α compared with control cells (Fig. 3D). We further depicted the type of ubiquitination that L3MBTL3 utilized to regulate HIF-1α. 293T cells were co-transfected with L3MBTL3 and plasmids containing only K48 or K63 ubiquitin respectively. The cells were then prepared to an in vivo ubiquitination assay, the results showed that L3MBTL3 promoted the HIF-1α K48-linked ubiquitination (Fig. 3E). Collectively, these data demonstrate that L3MBTL3 promotes the ubiquitination-dependent proteasomal degradation of HIF-1α.

### 3.4 L3MBTL3 makes an interaction with HIF-1α inside nucleus

**Fig. 4:**
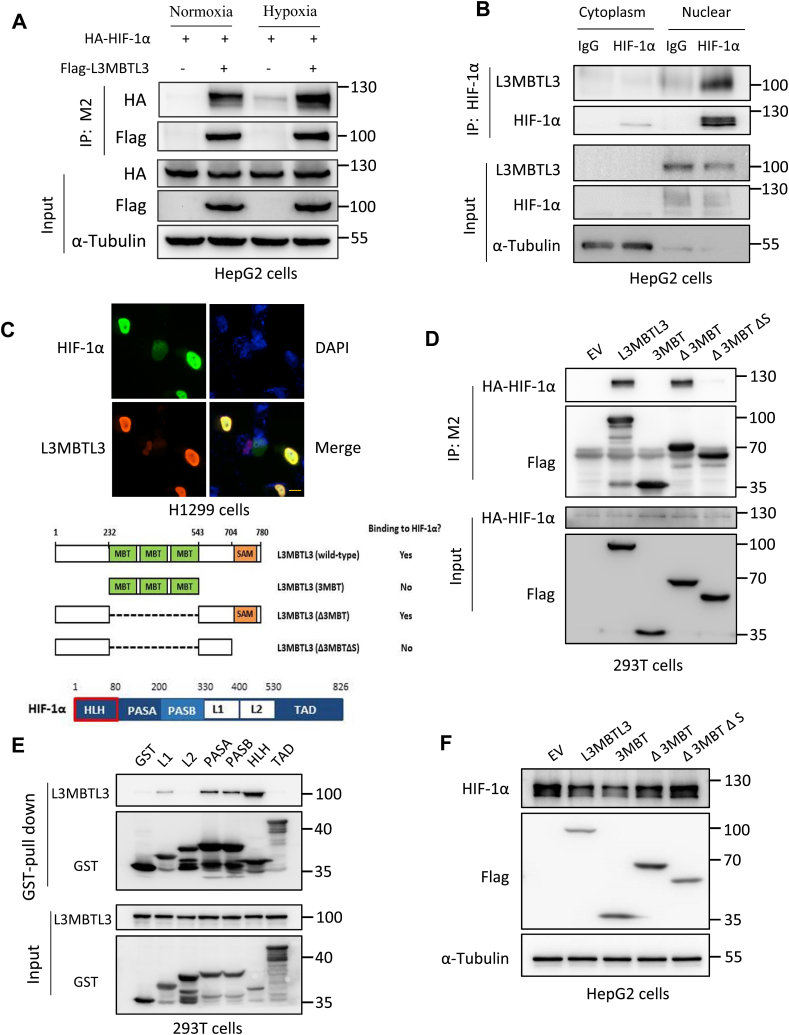
L3MBTL3 interacts directly with HIF-1α inside the cell nucleus A. Mutual interaction between exogenous HIF-1α and L3MBTL3 was explored. HepG2 cells transfected with Flag-L3MBTL3, HA–HIF–1α or control plasmids were cultured in normoxic or hypoxic states for 12 h. The cell lysates were then undergone immunoprecipitation analysis with anti-Flag beads. B. The interaction of endogenous HIF-1α and L3MBTL3 appeared inside the nucleus. Hypoxia cultured HepG2 cells were harvested followed by nucleus and cytoplasm separation. The different part lysates were then gone through immunoprecipitation assay with IgG, *anti*-L3MBTL3, *anti*–HIF–1α antibodies. C. Immunostaining showing that the colocalization of HIF-1α (green) and L3MBTL3 (red) in the nucleus (blue), scale bar: 10 μm. D. SAM domain of L3MBTL3 interacted with HIF-1α. 293T cells were transfected with HA–HIF–1α plasmids together with the empty vector control, plasmids encoding wild-type L3MBTL3 or mutants with deletion of different functional domains (right). The anti-Flag (M2) beads were then utilized to do the cells lysate immunoprecipitation assay. E. The interaction between L3MBTL3 and HIF-1α mainly relied on HLH domain. The purified bacteria expressed GST protein or HIF-1α GST-tagged truncations were made mixture with 293T cell lysates. The exogenous L3MBTL3 were immunoprecipitation with *anti*-GST beads then subjected to GST-pull down. F. The 3MBT domain of L3MBTL3 reduced the stability of HIF-1α. The raw figures for Western blot were shown in the Supplementary Figure S4.

We then analyzed if L3MBTL3 and HIF-1α could interact with each other by performing Co-IP assays. Normoxia or hypoxia cultured (12 h) HepG2 cells transfected with control vector or Flag tagged L3MBTL3 construct together with HA–HIF–1α plasmids. We observed co-precipitations of Flag-L3MBTL3 and HA–HIF–1α in normoxia and hypoxia cells (Fig. 4A). To further identify cellular localization of their interaction, the cytosol and nucleus were separated and isolated from HepG2 cells treated with hypoxic conditions. Co-IP assays revealed that L3MBTL3 made an interaction with HIF-1α mainly inside the nucleus (Fig. 4B). The immunostaining performed in the H1299 cells under hypoxic conditions revealed the colocalization between HIF-1α and L3MBTL3 in the nucleus (Fig. 4C).

L3MBTL3 contains a 3MBT domain with three MBT repeats, a putative phenylalanine-cysteine-serine nucleic acid−binding domain (FCS), a zinc finger and a sterile α motif (SAM) domain [26]. MBT domains are known to mediate binding to methylated lysine residues within histone tails [27]. SAM domain (Sterile Alpha Motif) has been shown to form helical polymer structures and mediate protein-protein interactions [[28], [29], [30]]. To identify the domains in L3MBTL3 that had an interaction with HIF-1α, we obtained a succession of L3MBTL3 mutant fragments with Flag tags. These constructed fragments and empty vector together with HA–HIF–1α plasmids were transfected into 293T cells. The immunoprecipitation using Flag antibody (M2) beads showed that the SAM domain of L3MBTL3 was required to the interaction with HIF-1α (Fig. 4D). To determine the protein regions in HIF-1α associated with L3MBTL3, a series of HIF-1α fragments as shown in Fig. 4D were expressed by bacteria. The same amount of each fragment and the cell lysates of 293T were made mixture individually. GST-pull-down assays gave evidence that the L1, PASA, PASB and *N*-terminal containing HLH regions of HIF-1α interacted with L3MBTL3 with the HLH region being the strongest one (Fig. 4E).

We then determined which fragment(s) of L3MBTL3 could mediate downregulation of HIF-1α. We transfected HepG2 cells with the control and Flag-tagged L3MBTL3 fragments plasmids and treated them with hypoxic conditions for 6 h. Western blot analysis showed that the 3MBT domain of L3MBTL3 could reduce the HIF-1α stability (Fig. 4F). Overall, the data above indicated that L3MBTL3 interacted with HIF-1α in the nucleus through its SAM domain, but downregulated HIF-1α through its 3MBT domain.

## 4 Discussion

Being a crucial transcription factor accountable for the cells and tissues adaptation to hypoxia, HIF-1α also helps tumor cells survive and progress in the hypoxic microenvironment through transcriptional activation of over 100 downstream genes [31]. Thus, timely turning down the activated HIF-1α is a critical step in HIF-1α signaling to avoid the detrimental effects of uncontrolled expression of hypoxia-inducible genes. In this in vitro study, we identified a novel negative regulator of HIF-1α, L3MBTL3.

As with many transcription factors critical in stress responses, suppression of transactivation activity and induction of degradation are two prime approaches to downregulate HIF-1α, which often involve a negative feedback mechanism. We show in this study that the transcription of L3MBTL3 is increased substantially by HIF-1α in hypoxic conditions, which can come into contact with HIF-1α and mediates its degradation through ubiquitination in the nucleus. However, since L3MBTL3 contains a methyl-lysine reader domain which has been shown to mediate the binding to methylated lysine residues within histone tails [26,32], we cannot rule out the possibility that L3MBTL3 might also regulate the transcriptional activity of HIF-1α through an epigenetic mechanism.

It is not clear how L3MBTL3 regulates the stability of HIF-1α. L3MBTL3 has been reported to be a crucial mediator in a methylation coupled ubiquitination system in which methylated factors, such as SOX2, DNMT1 and E2F1, are ubiquitinated and degraded. In this system, L3MBTL3 simultaneously binds a methylated protein and an E3 ubiquitin ligase complex through its methyl-lysine reader domain and a unique region between the zf-c2 and the SAM domain, respectively [22,23]. Several reports have demonstrated that there exists a HIF-1α methylation dependent ubiquitination and degradation mechanism that contributes to the degradation of HIF-1α in hypoxia [17,33]. In addition, it is reported that L3MBTL3 associated methylated Bclaf1, which has been shown to form a complex with HIF-1α [24,32]. Thus, it is tempting to speculate that L3MBTL3 mediated HIF-1α degradation might also involve HIF-1α methylation.

In conclusion, L3MBTL3 is a critical component in a negative feedback loop in the HIF-1α signaling that mediates HIF-1α degradation in hypoxia.

## Author contribution statement

Mengdong Wang, Di Wang: Performed the experiments; Analyzed and interpreted the data; Wrote the paper.

Yue Lang: Analyzed and interpreted the data; Contributed reagents, materials, analysis tools or data.

Anwen Shao, Rui Zhang: Analyzed and interpreted the data.

Jun Tang: Designed the project and made manuscript revisions.

Jun Tang, Dongming Lai: Conceived and designed the experiments; Wrote the paper.

Chenglu Xiao: Conceived and designed the experiments; Performed the experiments; Wrote the paper.

## Funding statement

Rui Zhang and Jun Tang were supported by 10.13039/501100001809National Natural Science Foundation of China [32172829 & 32072848].

## Data availability statement

No data was used for the research described in the article.

## Declaration of interest's statement

The authors declare that they have no known competing financial interests or personal relationships that could have appeared to influence the work reported in this paper.
