# Supplementary material for: L3MBTL3 is induced by HIF-1α and fine tunes the HIF-1α degradation under hypoxia in vitro
Source: Heliyon. 2023 Jan 24;9(2):e13222. doi: 10.1016/j.heliyon.2023.e13222 (PMC9898070; doi:10.1016/j.heliyon.2023.e13222)

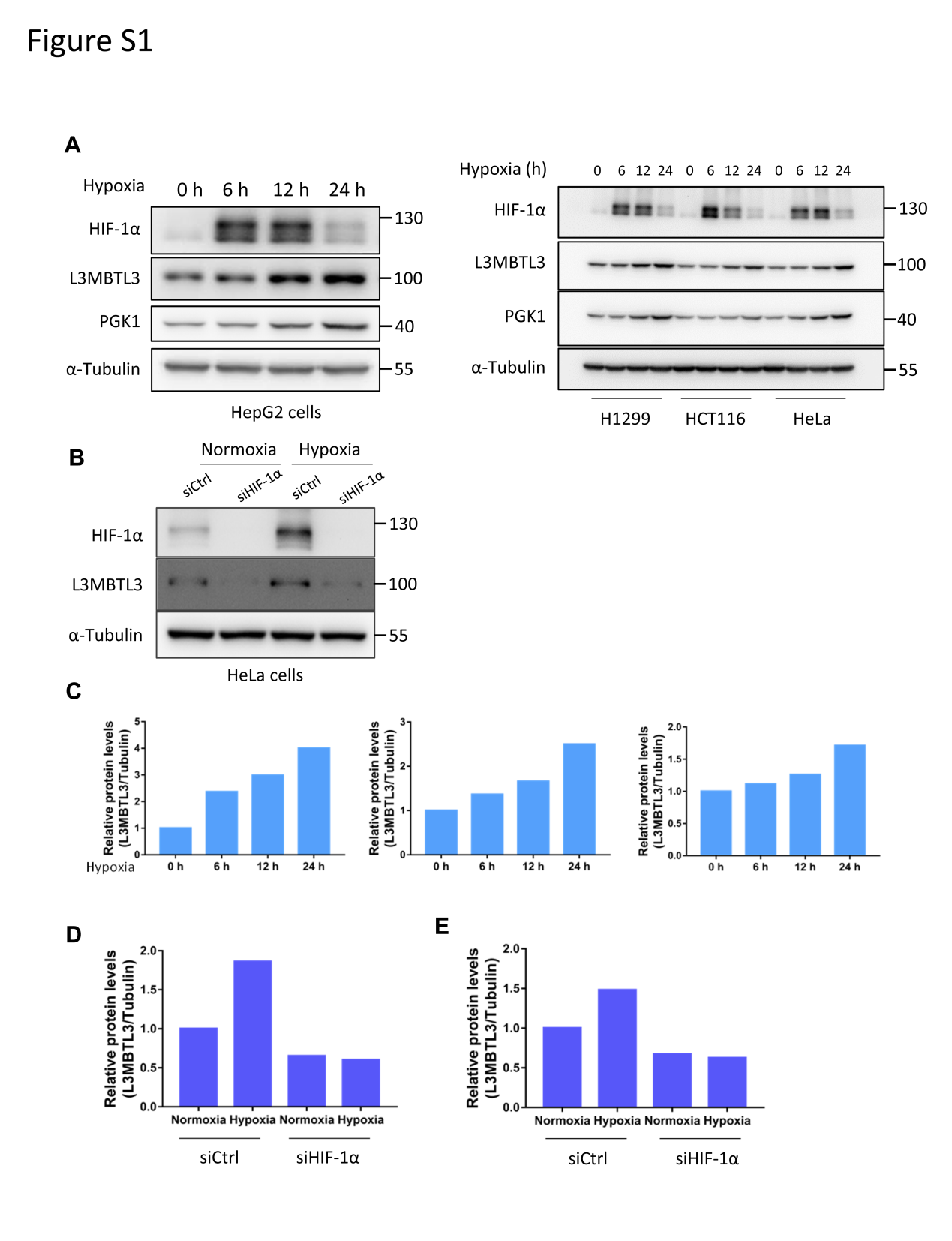


A. HepG2, H1299, HCT116 and HeLa cells were treated with low oxygen conditions (1% O_2_) for the displayed time points. The cells lysates were analyzed by Western blot using the indicated antibody. B. In respect of normoxia or hypoxia , Western blot analysis of HeLa cells transfected with siRNA of control or HIF-1α. C-E. The relative intensity of the bands of L3MBTL3 to α-Tubulin was analyzed by Image J software(for Figure 1A, 1B and 1E).


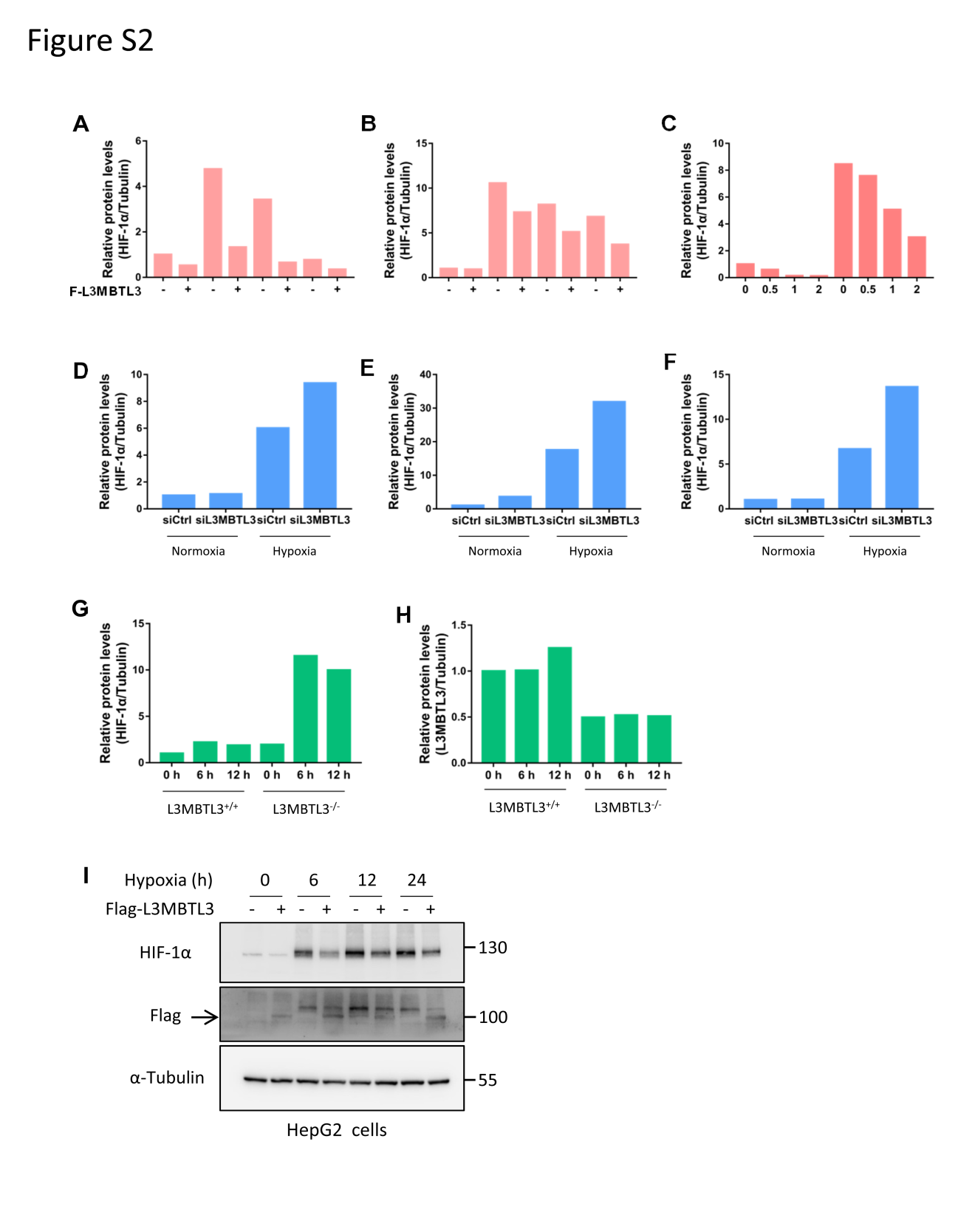


A-G. The relative intensity of the bands of HIF-1α to α-Tubulin was analyzed by Image J software(for Figure 2A-2G). H. The relative intensity of the bands of L3MBTL3 to α-Tubulin was analyzed by Image J software(for Figure 2G). I.HepG2 cells were transfected with control or Flag-L3MBTL3 plasmids, Western blot analysis was used to analyze the protein levels of HIF-1α.


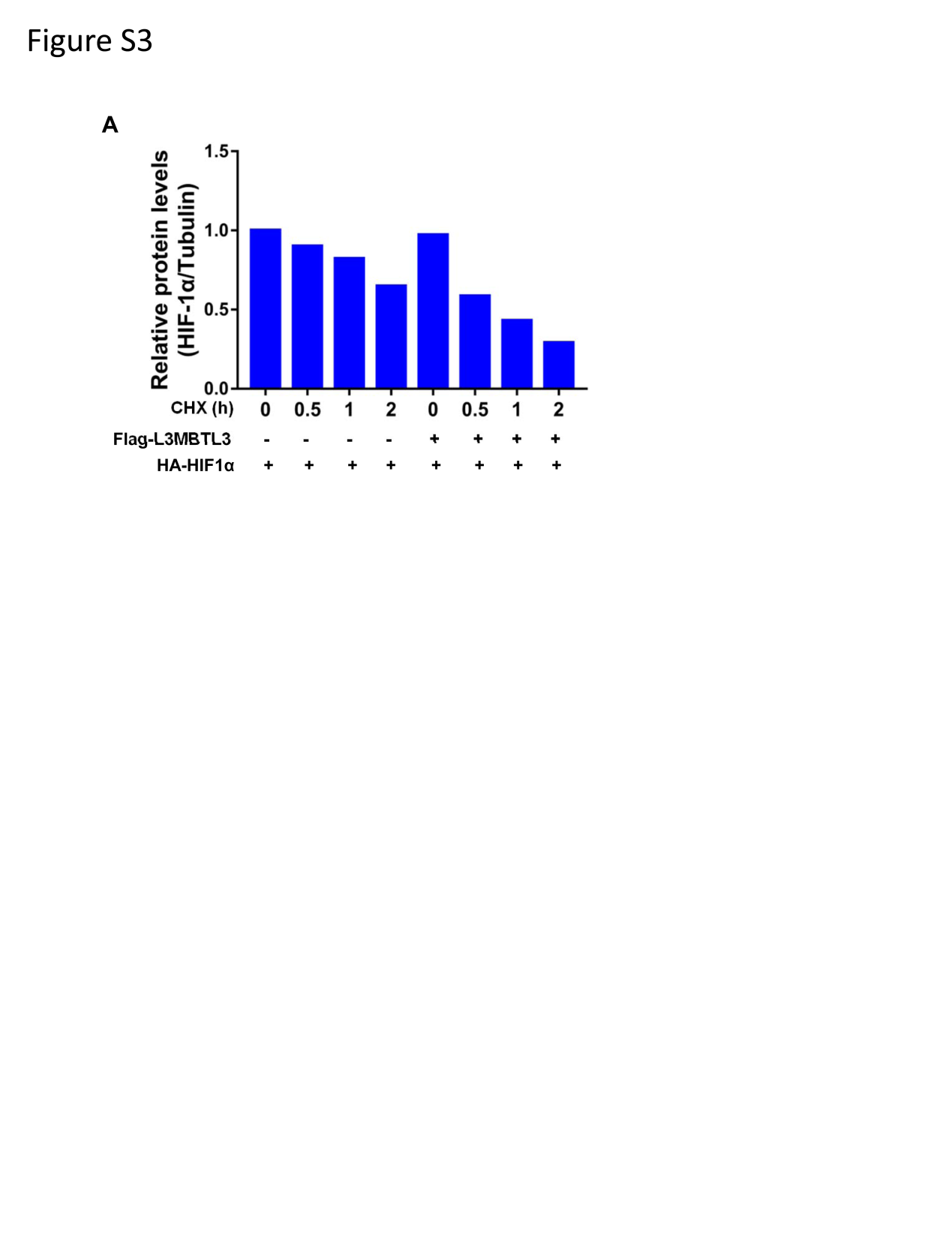


A. The relative intensity of the bands of HIF-1α to α-Tubulin was analyzed by Image J software(for Figure 3C).

Figure S4

**Raw figures for Figure 1**


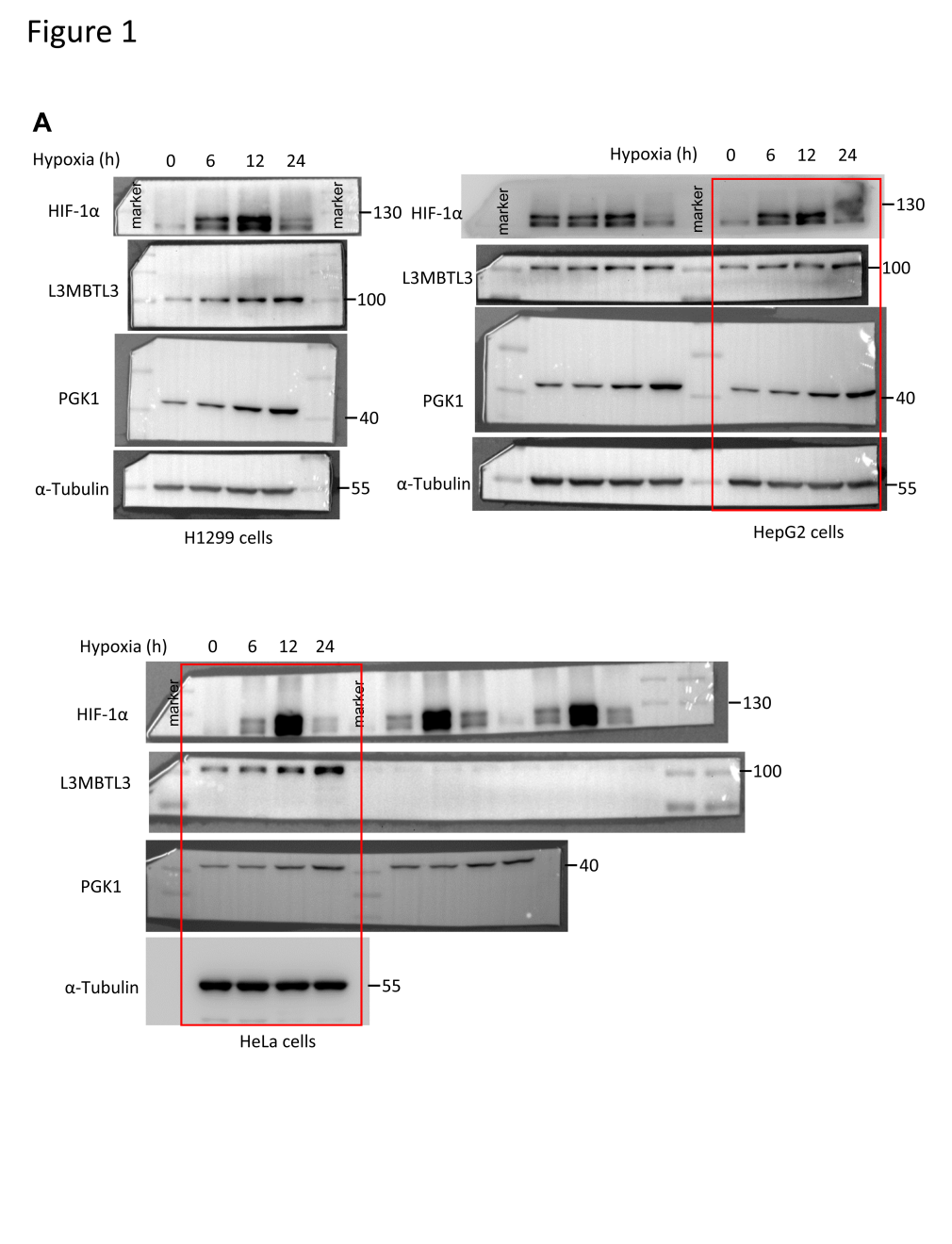


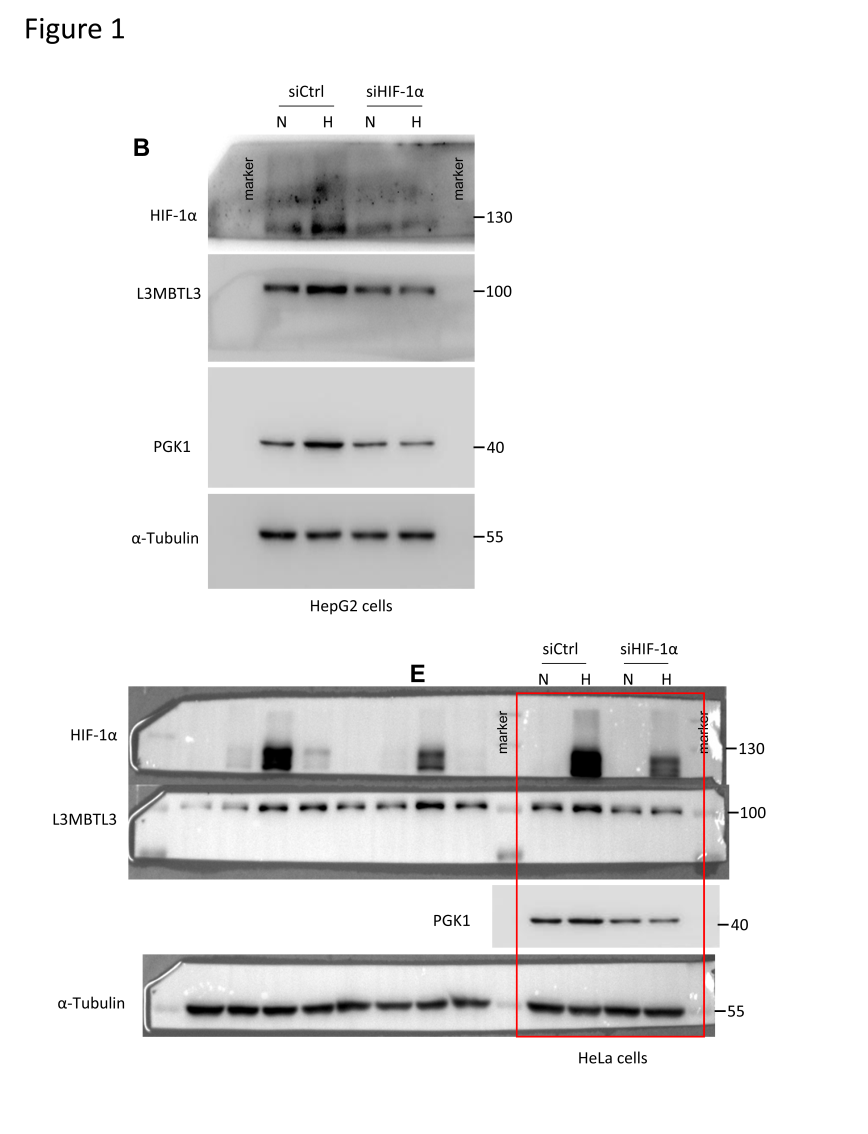


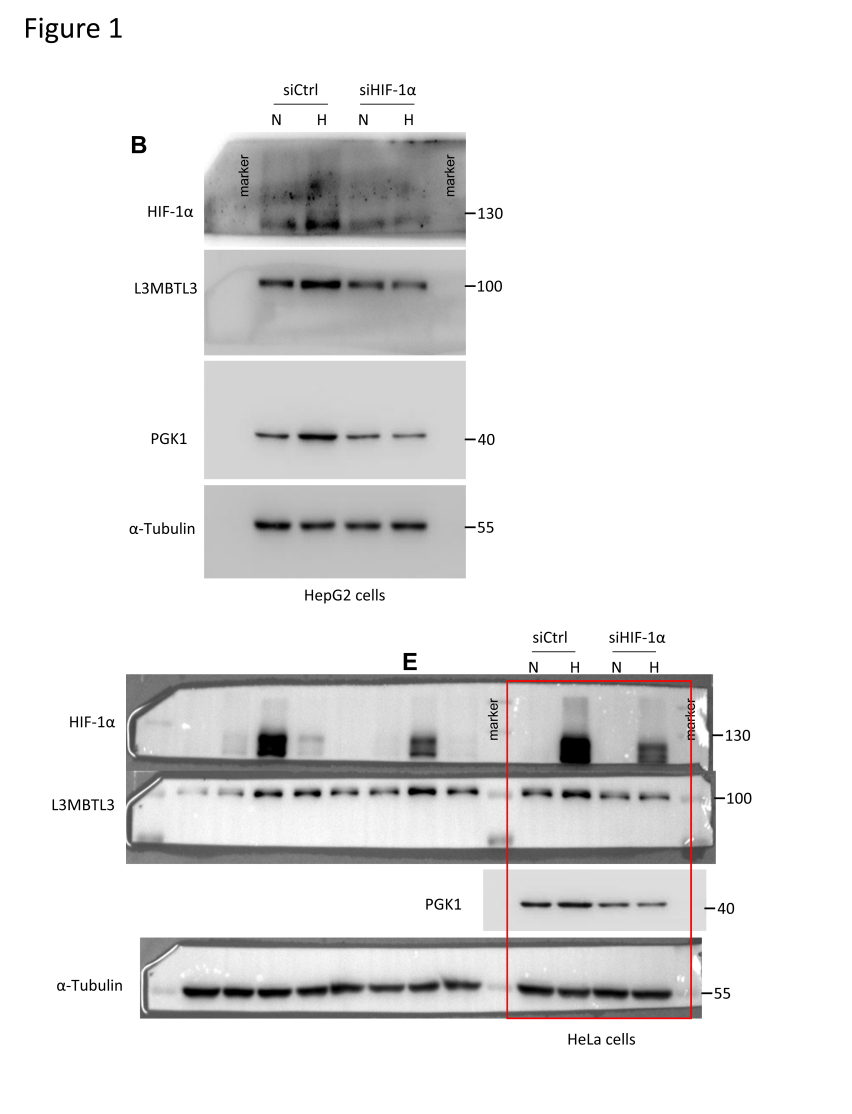


**Raw figures for Figure 2**


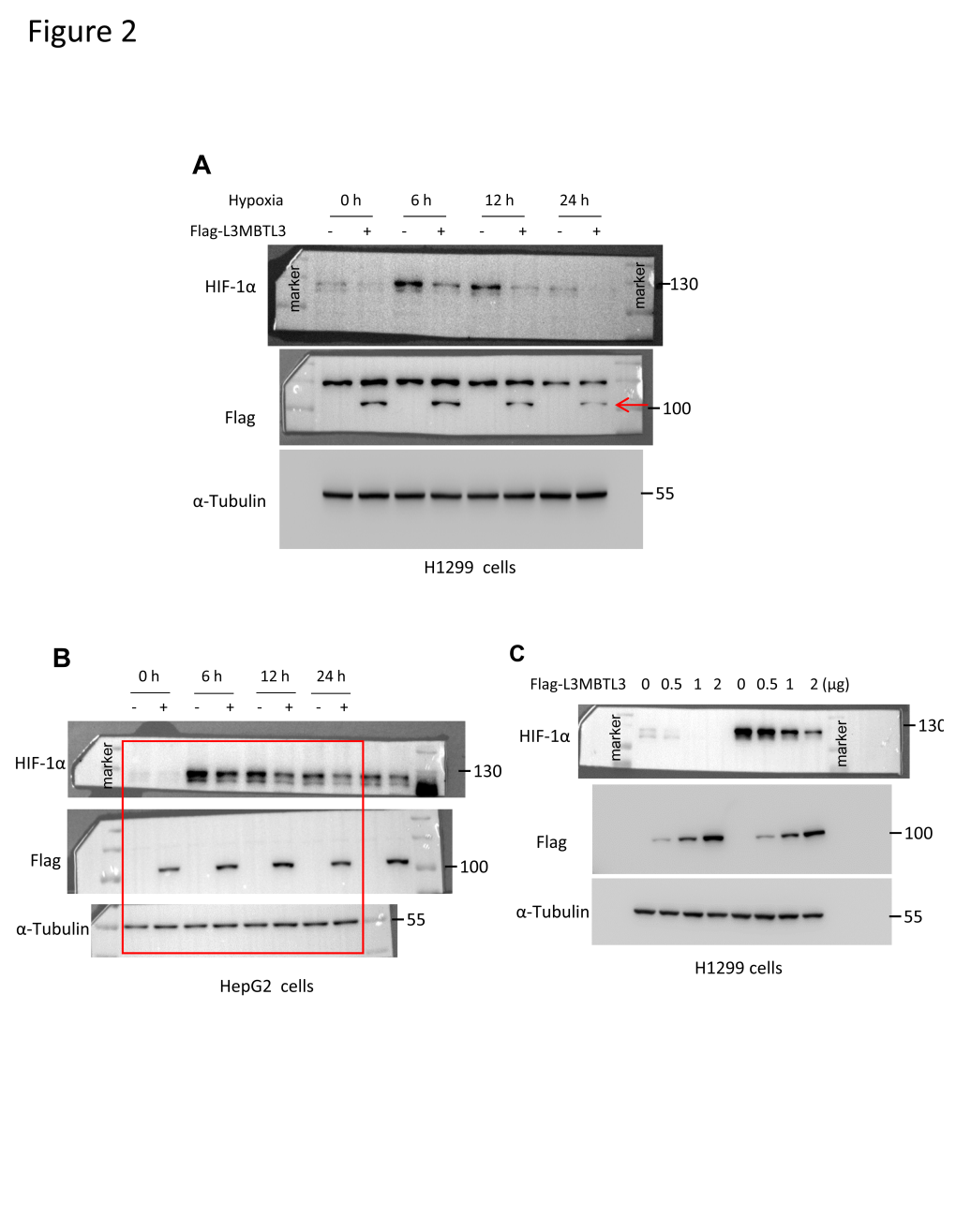


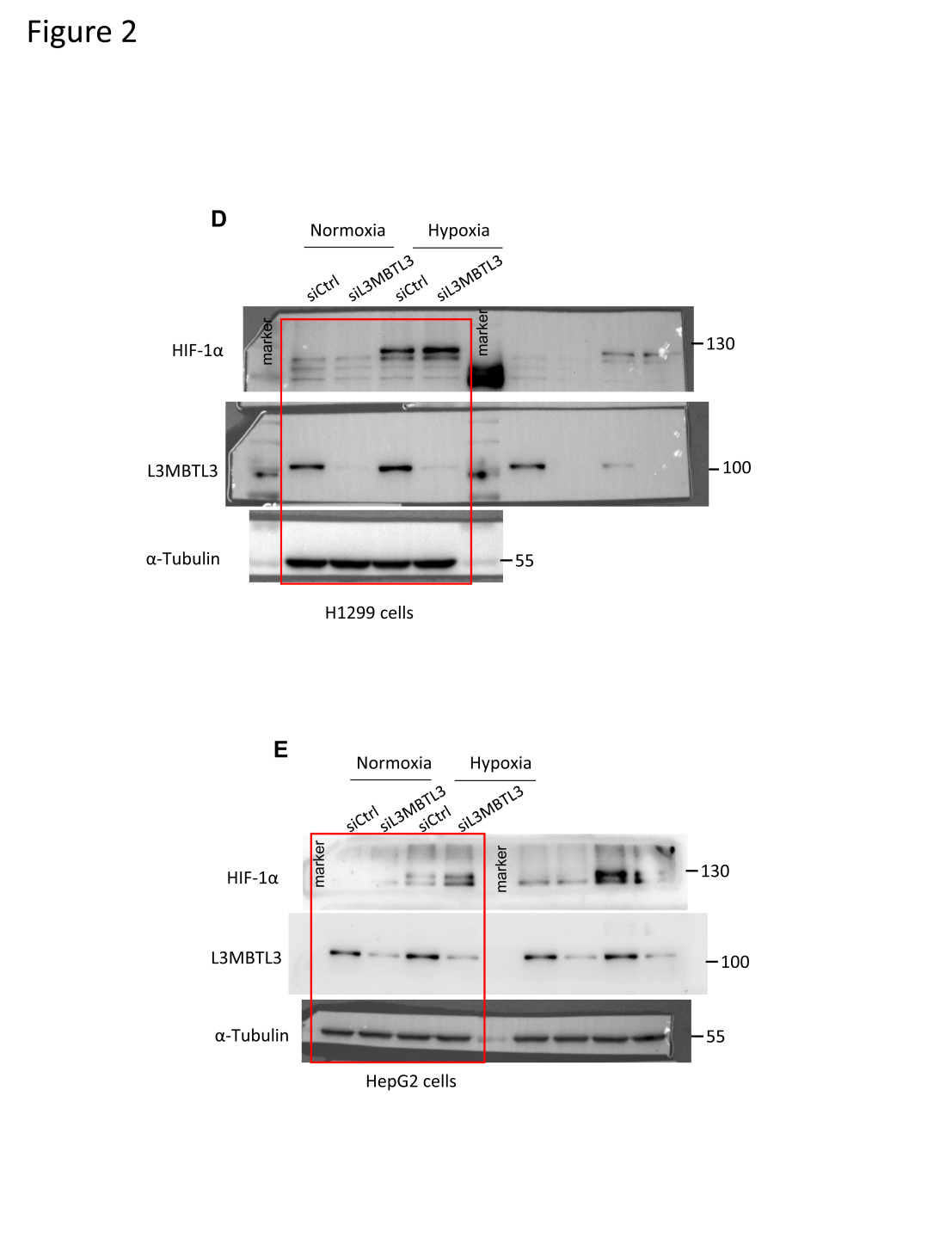

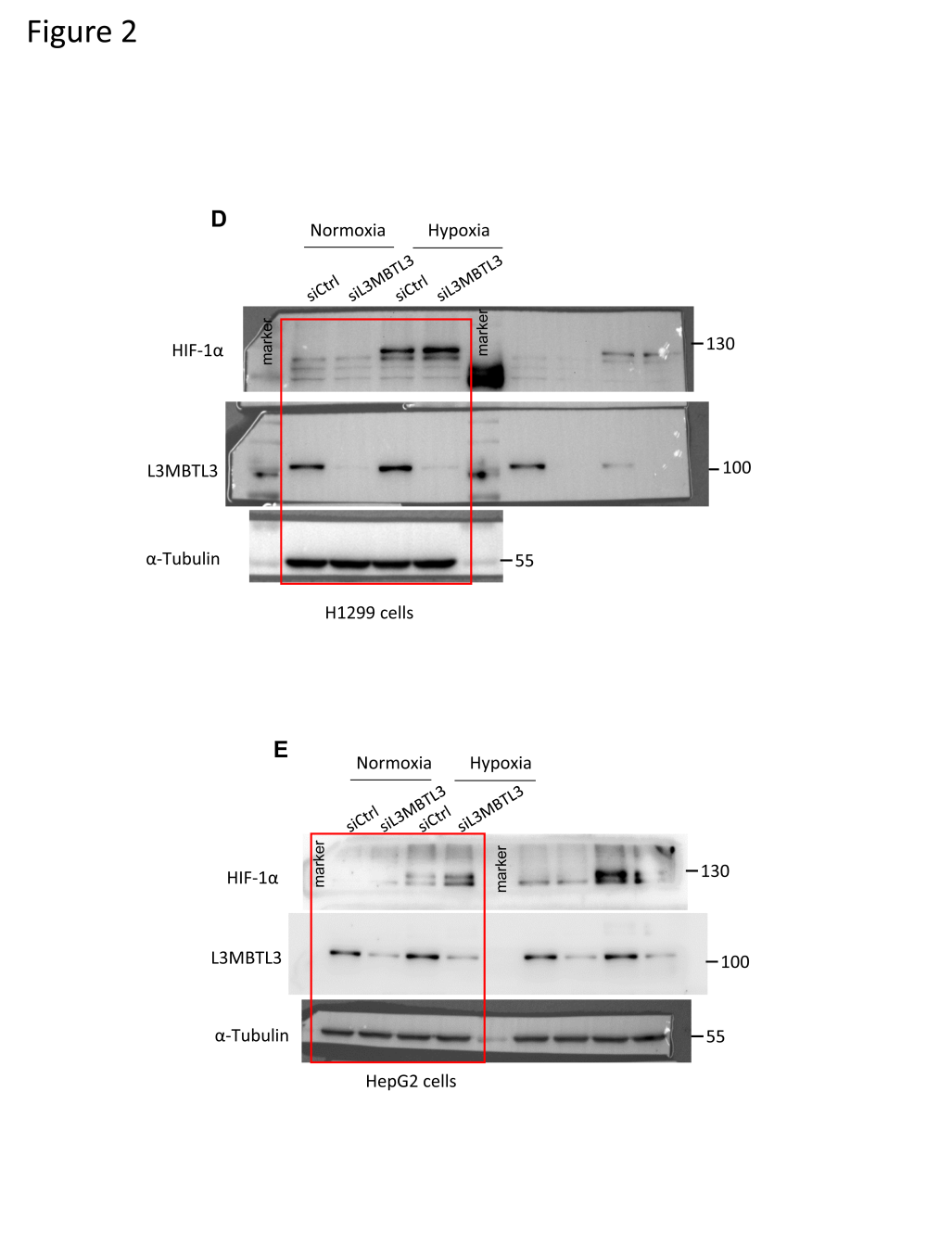


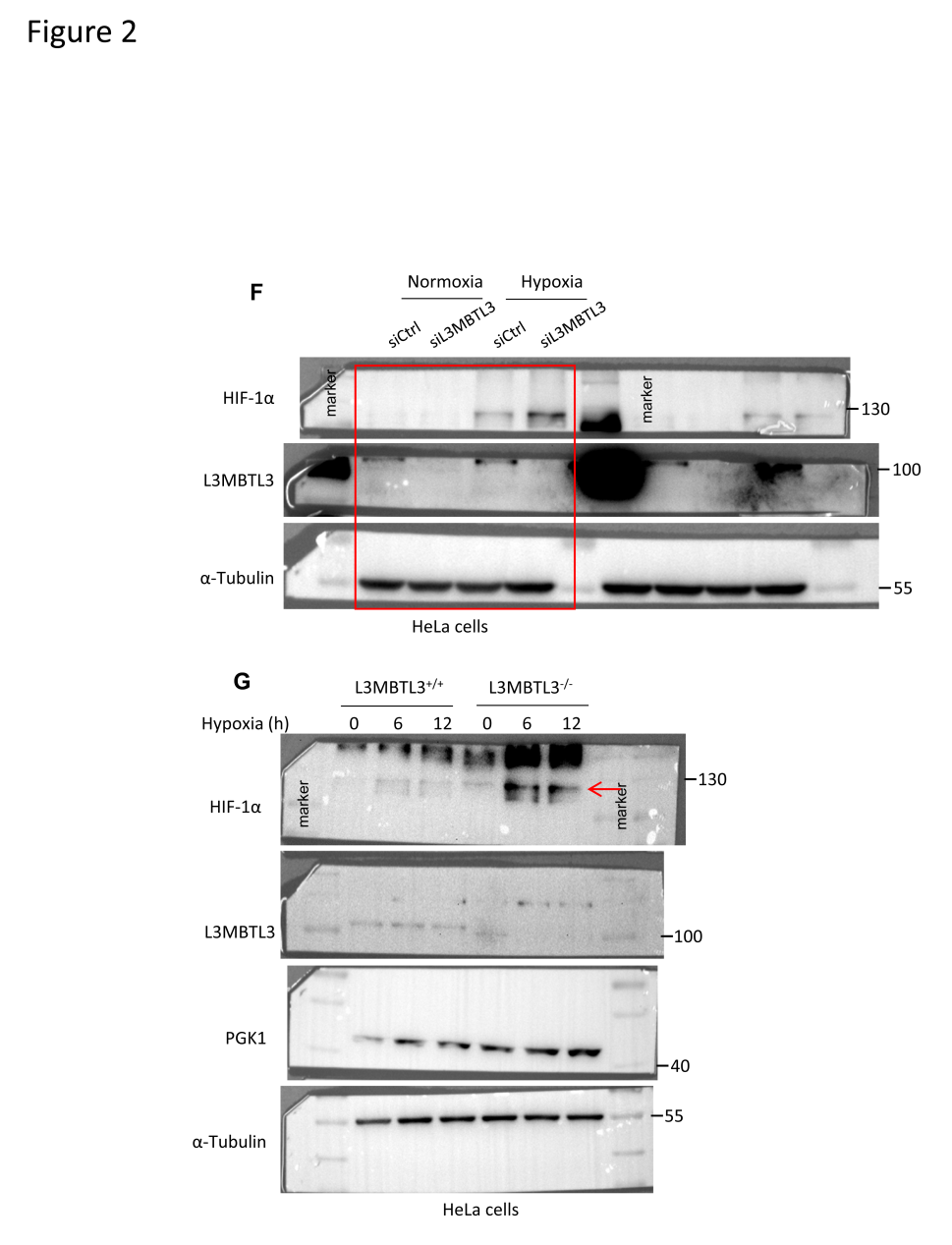


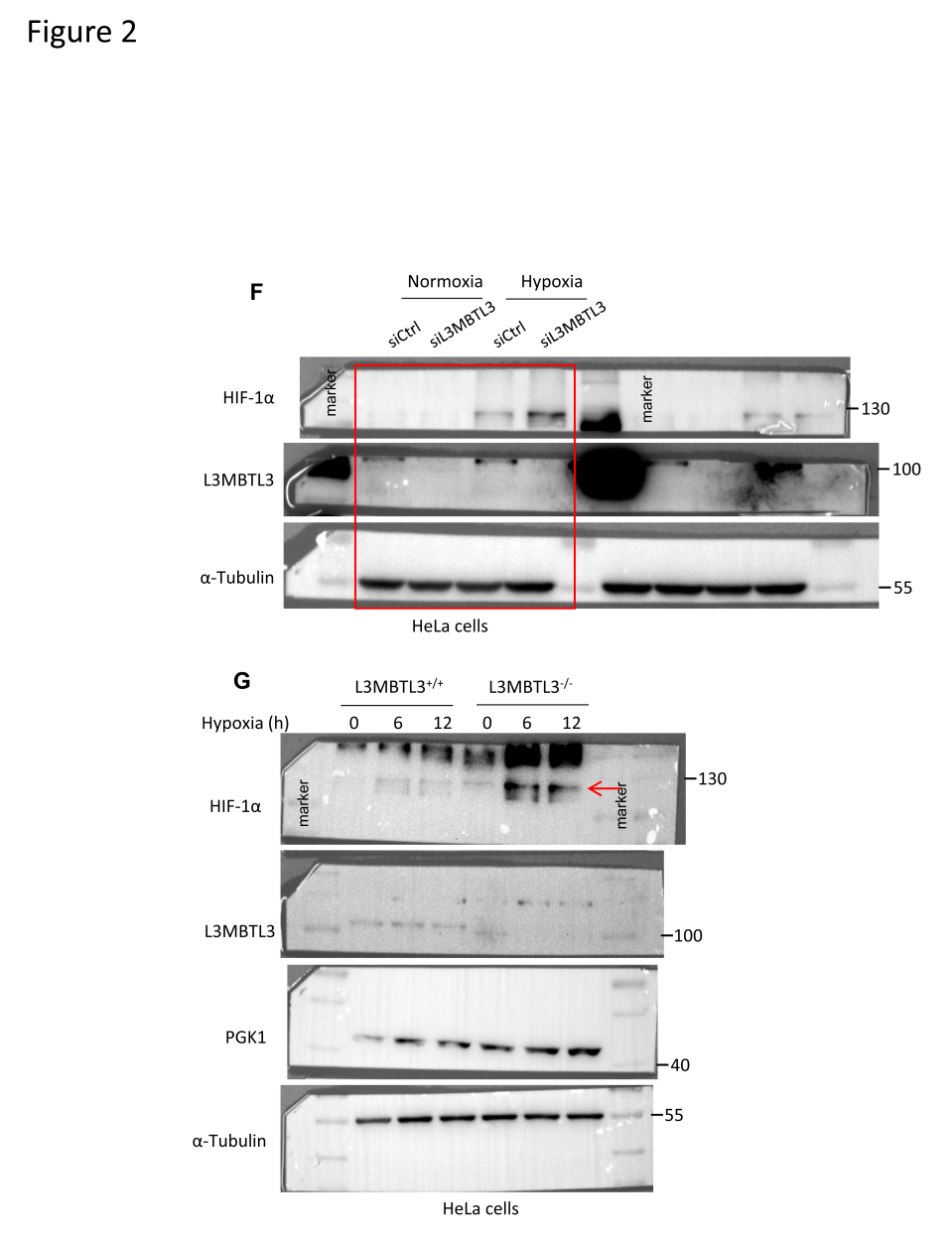


**Raw figures for Figure 3**


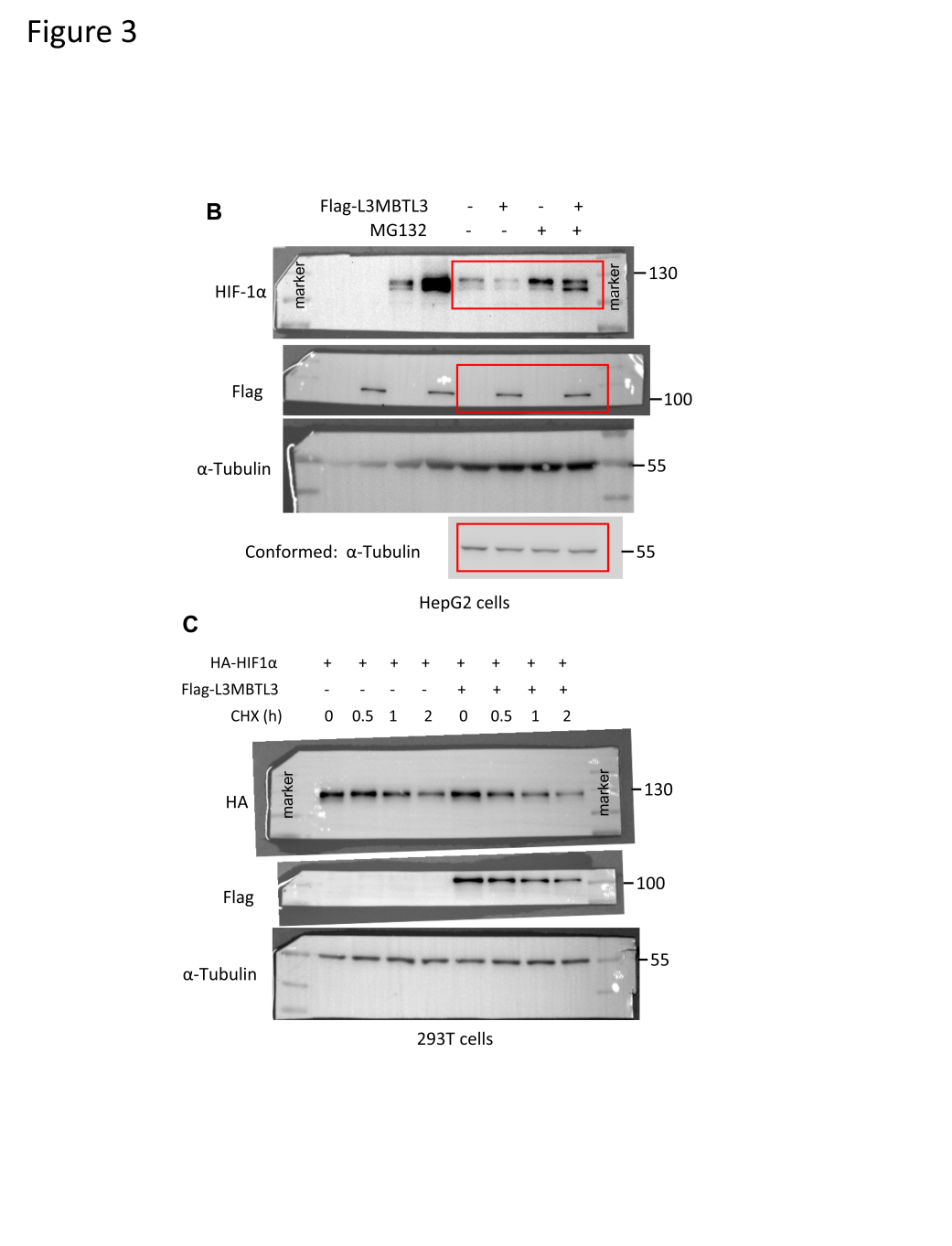

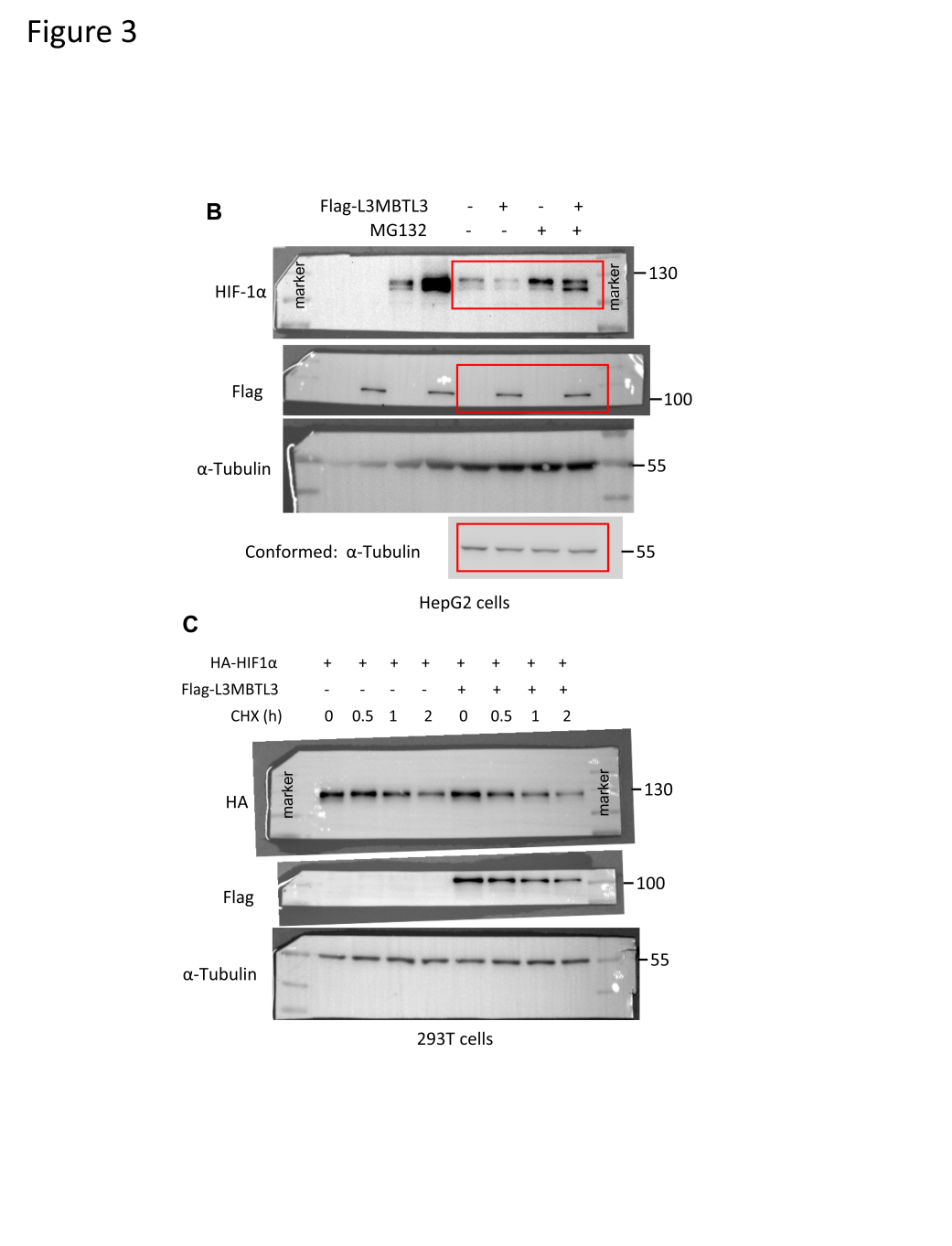


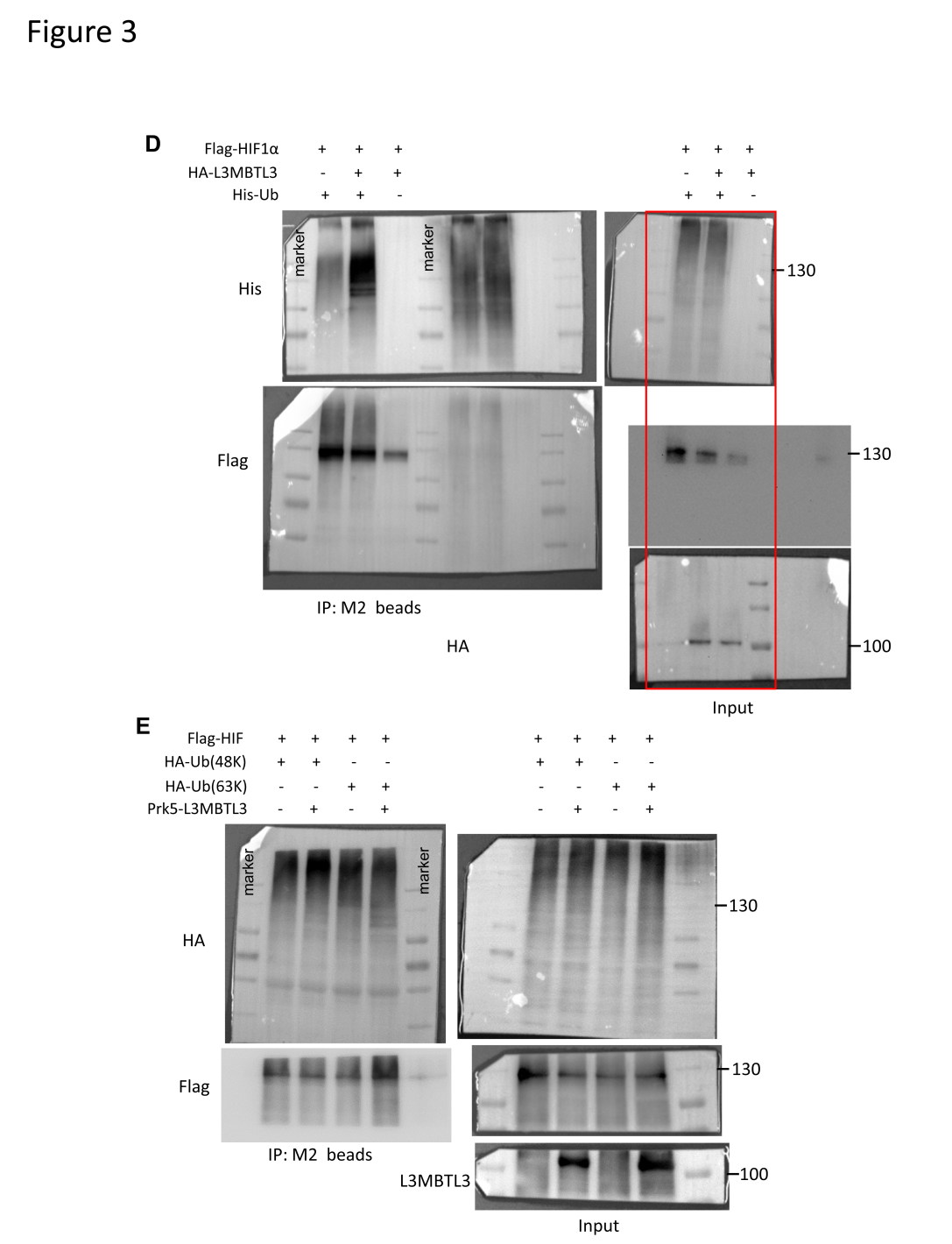


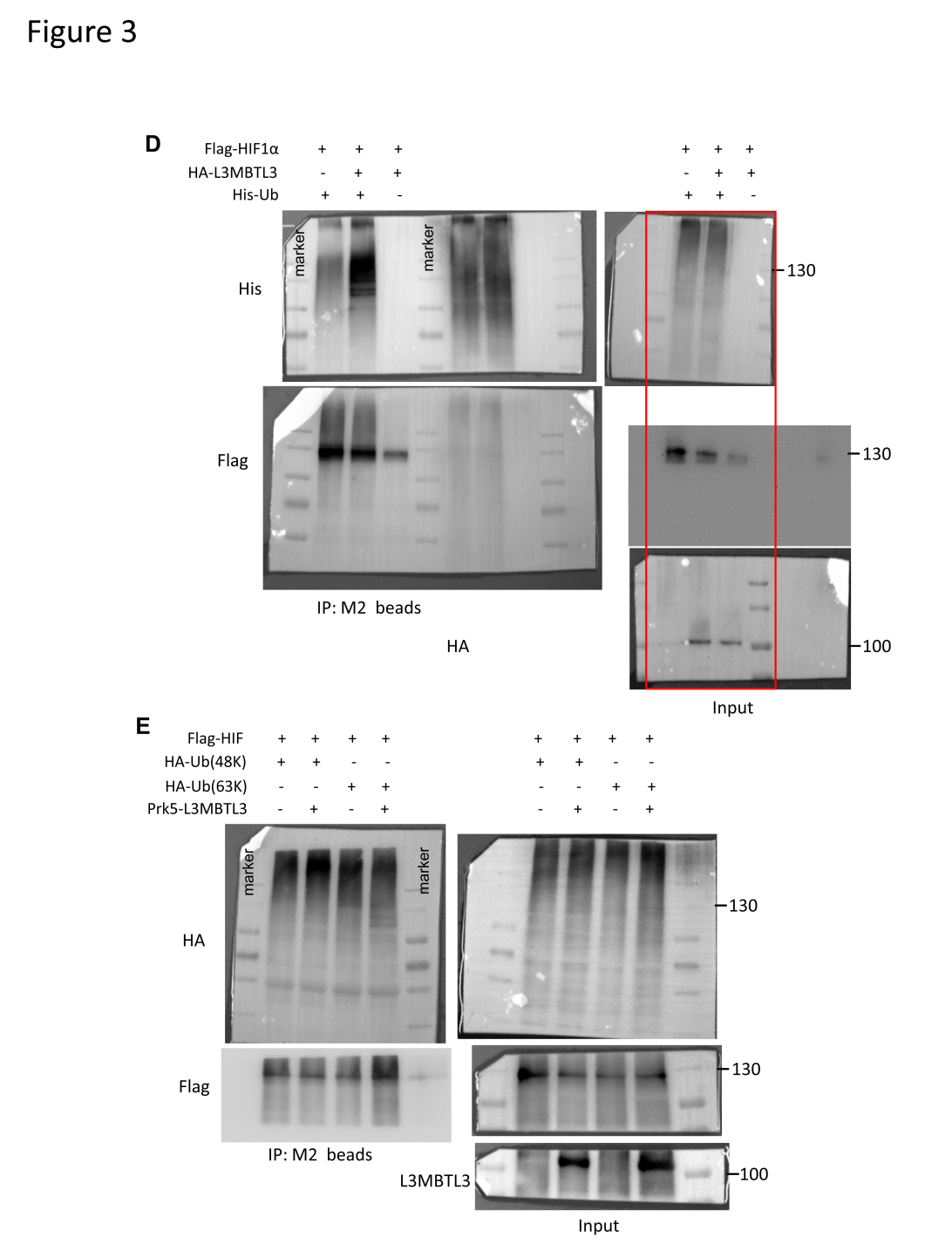


**Raw figures for Figure 4**


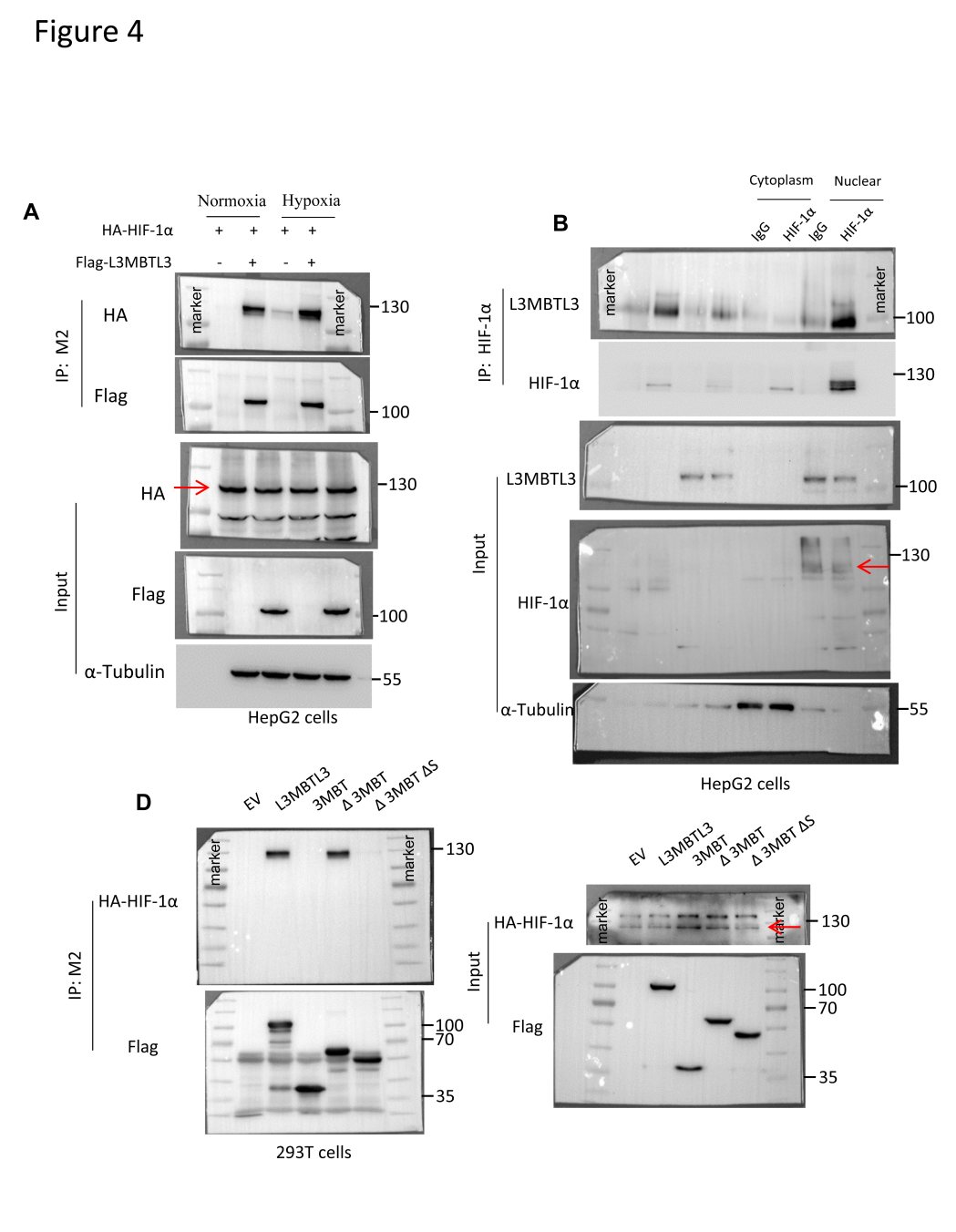


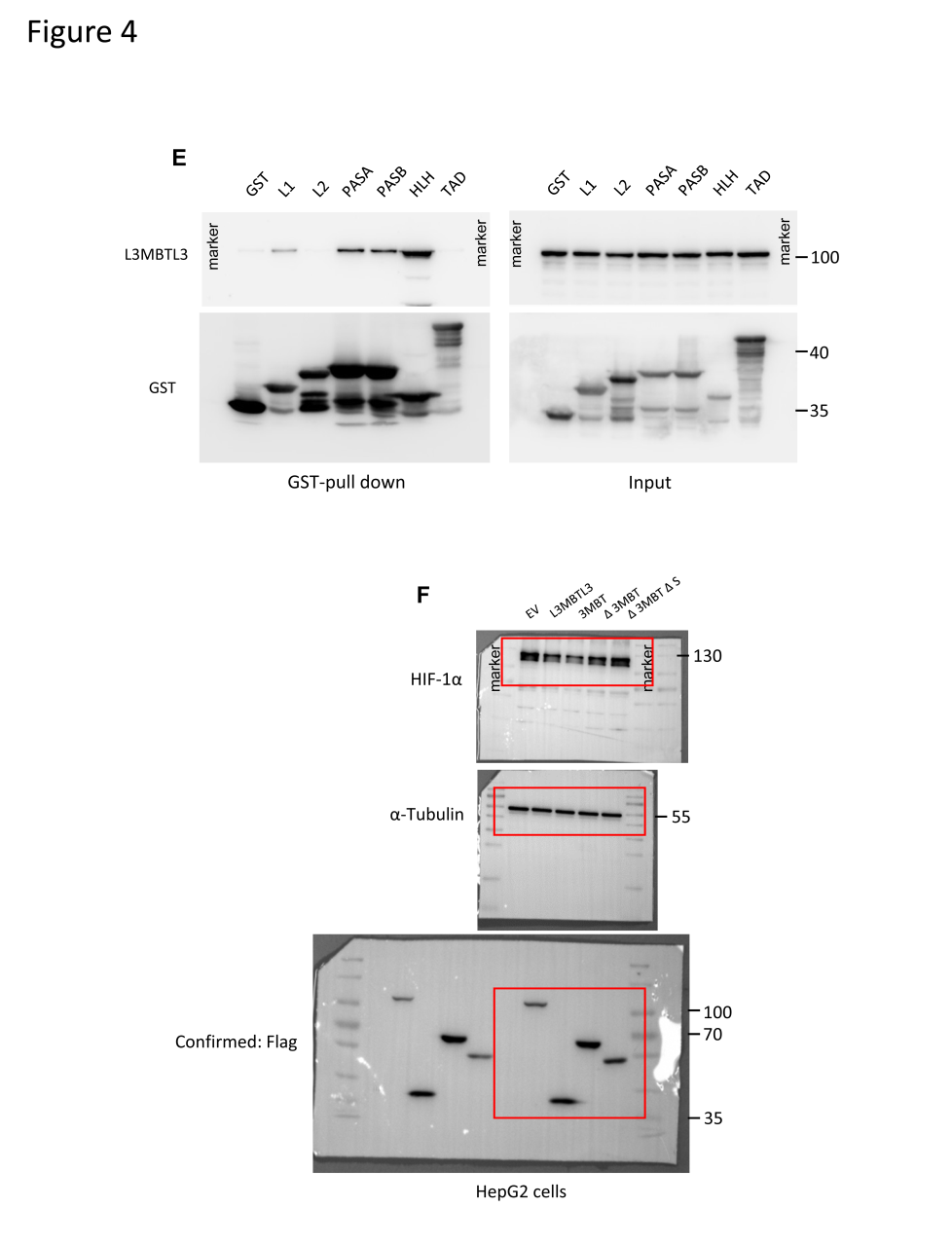


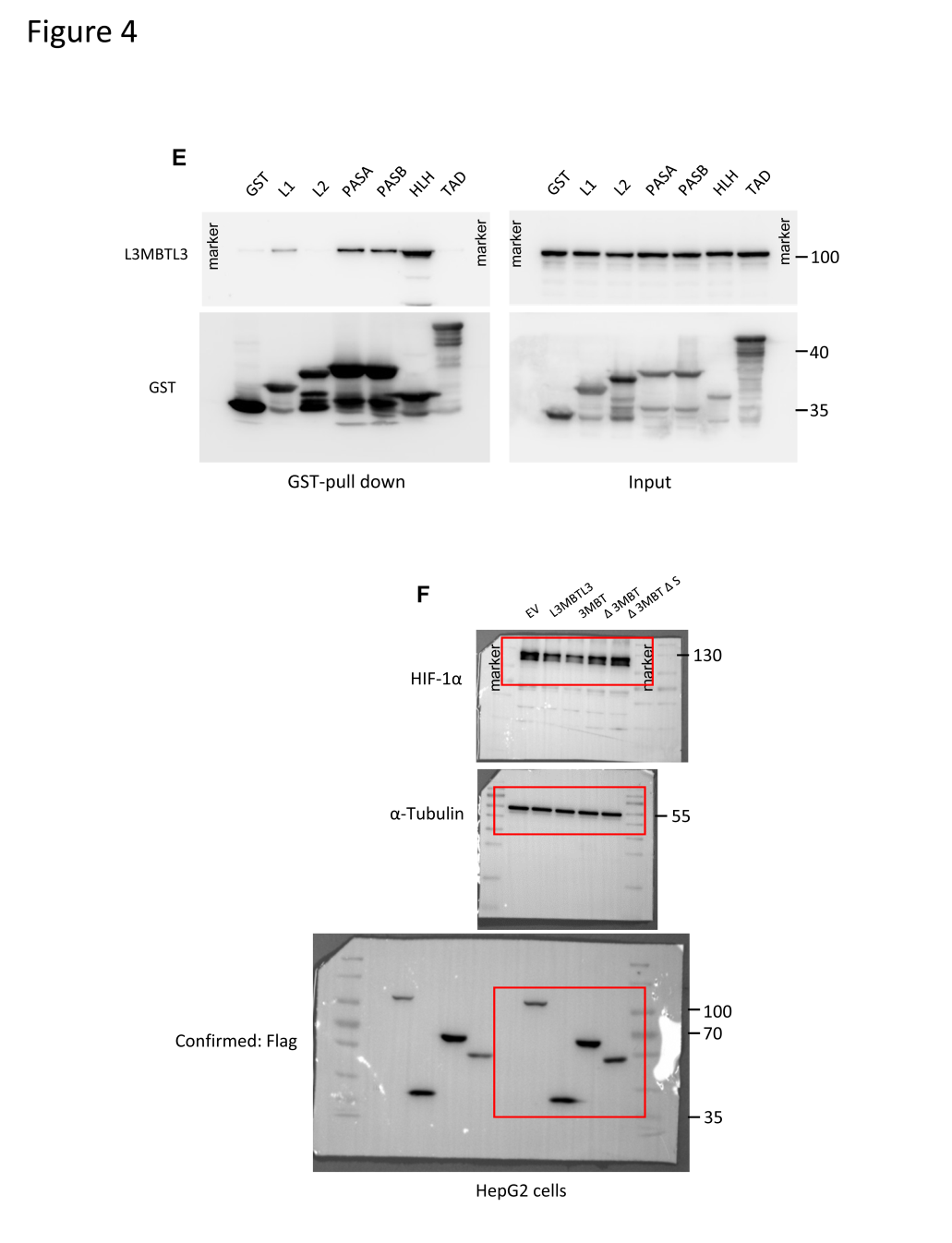

Supplement: Multimedia component 1 [file mmc1.docx]
